# Creutzfeldt–Jakob disease: A case report and differential diagnoses

**DOI:** 10.1002/ccr3.6239

**Published:** 2022-08-10

**Authors:** Akash Raut, Anjila Thapa, Ashish Shrestha, Kamal Saud, Reema Rajbhandari, Shailendra Katwal

**Affiliations:** ^1^ Maharajgunj Medical Campus Institute of Medicine Kathmandu Nepal; ^2^ Department of Neurology Tribhuvan University Teaching Hospital Kathmandu Nepal; ^3^ Department of Radiology Tribhuvan University Teaching Hospital Kathmandu Nepal

**Keywords:** neurodegeneration, psychoneuroimmunology, prion disease, sporadic Creutzfeldt–Jakob disease

## Abstract

Although sporadic Creutzfeldt–Jakob disease is a rare neurodegenerative disease and often difficult to diagnose at the earliest onset, meticulous clinical examination, electroencephalography, and neuroimaging findings will help in diagnosis.

## Graphical abstract

Creutzfeldt–Jakob disease (CJD) can also be diagnosed in a resource‐limited setting through proper and meticulous clinical analysis. Accurate diagnostic workup and management of patients with rapidly progressive dementia can be done even in countries with limited available tools and where CJD has never been reported before.

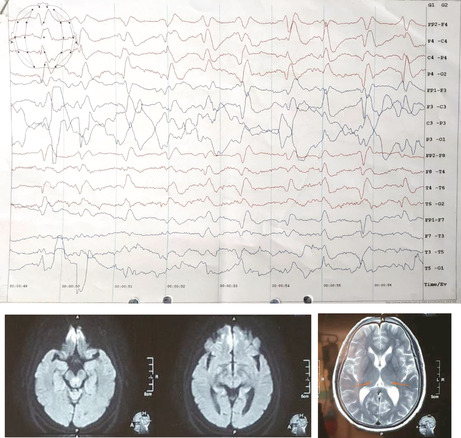

## 1 INTRODUCTION

Creutzfeldt–Jakob disease (CJD) is a rare and fatal human neurodegenerative disorder characterized by a rapidly progressive dementia, myoclonus, cerebellar, pyramidal, extrapyramidal, visual symptoms, and psychiatric manifestations.^1^ Sporadic CJD accounts for 90% of cases, while 5–10% are due to genetic CJD; Iatrogenic CJD and Variant CJD generally account for less than 1%.^2^, ^3^ Approximately 1 case of Sporadic CJD (sCJD) occurs per one million individuals across the entire population per year with a worldwide distribution.^3^

It is caused by the conversion of the normal form of prion protein (PrPC, prion‐related protein, in which C stands for the cellular form of the protein) with a primarily alpha‐helical structure into an abnormal form of the prion protein (PrPSc, proteinaceous infectious particle, in which Sc stands for scrapie, the prion disease of sheep and goats), which has a primarily beta‐pleated sheet structure, which then spreads and accumulates throughout the brain leading to spongiform neurodegeneration.^1^ The initial diagnosis of CJD is obscured by its heterogeneous clinical and laboratory presentation.

We present a case report that includes the first reported case of sporadic CJD (sCJD) infected with SARS CoV‐2 and also illustrate the complexity of diagnosing this disease in the early stages of a clinical course in resource‐limited settings.

## 2 CASE REPORT

A 65‐year‐old woman was referred to our hospital with a 3‐month history of behavioral, and personality changes along with progressive cognitive decline. She was in her usual state of health until 3 months ago when her family members noticed that she seemed to have easy forgetfulness and worsening functional impairment. The forgetfulness was initially related to daily care activities, dressing, and self‐care and later progressed to the extent of not recognizing family members. Later, the patient developed stiffness in the entire body and started experiencing visual hallucinations, such as seeing random people in her surroundings. In the 2 weeks prior to the emergency room visit, her symptoms rapidly progressed, to an extent that she was unable to ambulate. There was also new onset urinary and fecal incontinence. She does not smoke or consume alcohol, and there is no significant past medical, surgical, or psychiatric history. There is no family history of dementia or other neurological disorders. At the time of admission, she was mute and appeared ill. Tone was increased, and brisk reflexes present across all extremities. Jaw jerk was prominent, and bilateral palmomental reflex was present. Continuous myoclonic jerks were noted involving all extremities. She was unable to ambulate secondary to worsening ataxia. Presence of forced eye gaze deviation was noticed at times. Paratonia in both upper limbs and lower limb and bilateral extensor plantar response.

**FIGURE 1:**
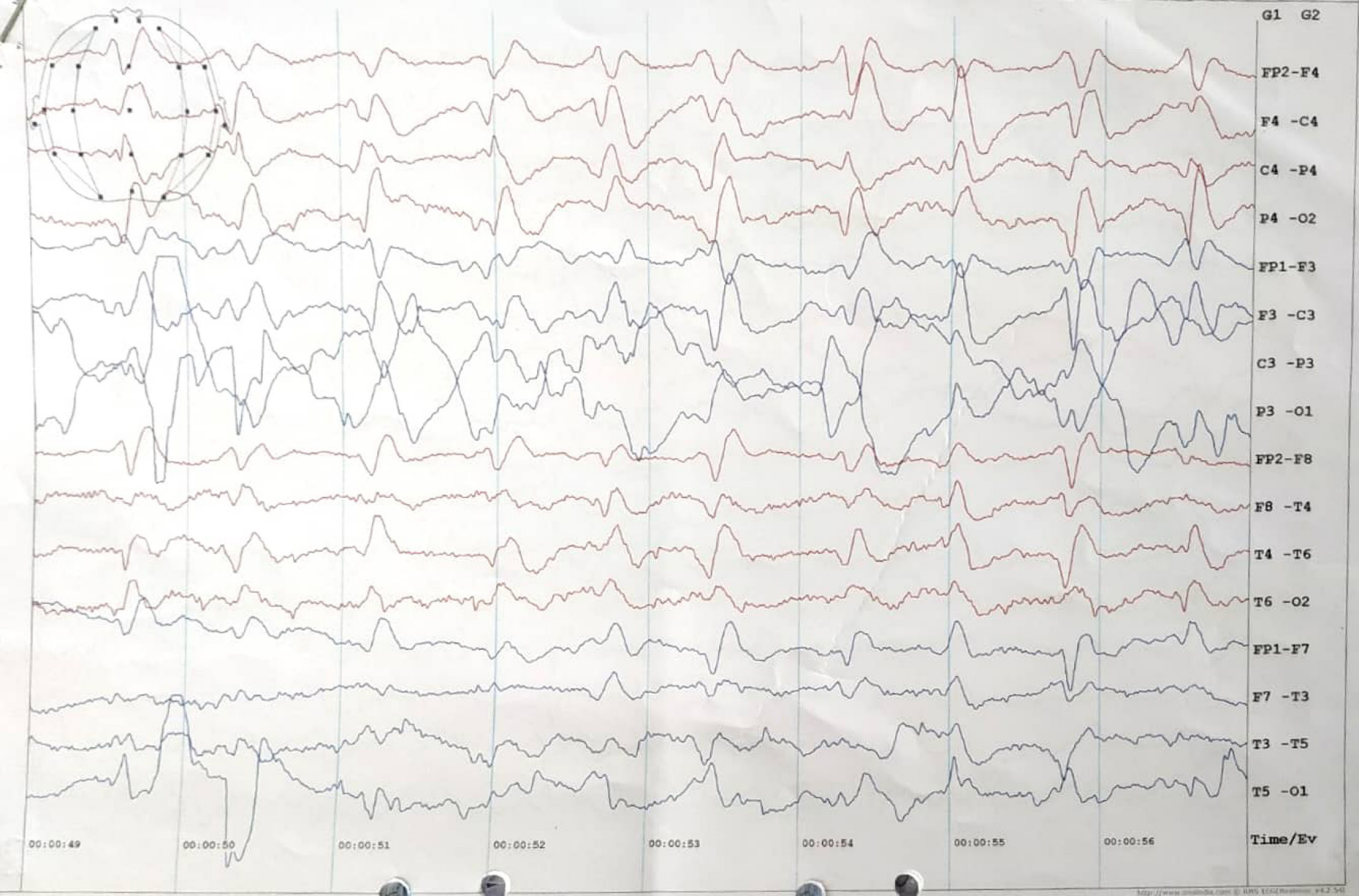
EEG OF BRAIN showed continuous periodic complexes and generalized slow waves as shown in this figure.

Routine laboratory investigations including serology for hepatitis B, HIV, and syphilis were all negative. Lumbar puncture for CSF evaluation along with culture revealed insignificant findings (total count: 2/cumm with only lymphocytes, sugar: 3.5 mmol/L and total protein 62 mg/dL, and ADA levels: 2.32 U/L). Thyroid hormones, vitamin B12, and folate levels were all within normal limits. Chest X‐ray, electrocardiogram, and abdomino‐pelvic ultrasound were otherwise unremarkable. EEG report showed continuous periodic complexes and generalized slow waves as shown in Figure 1.

**FIGURE 2:**
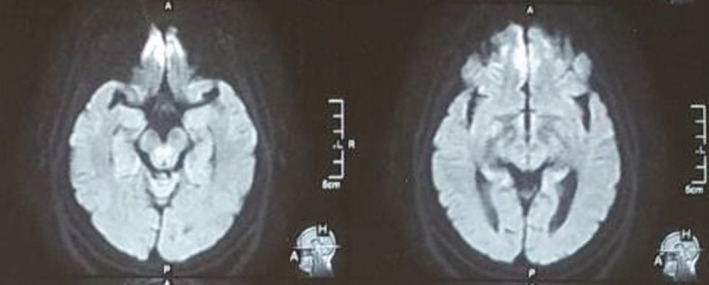
DWI high signal intensity in bilateral frontal (right> left) and occipital cortex

**FIGURE 3:**
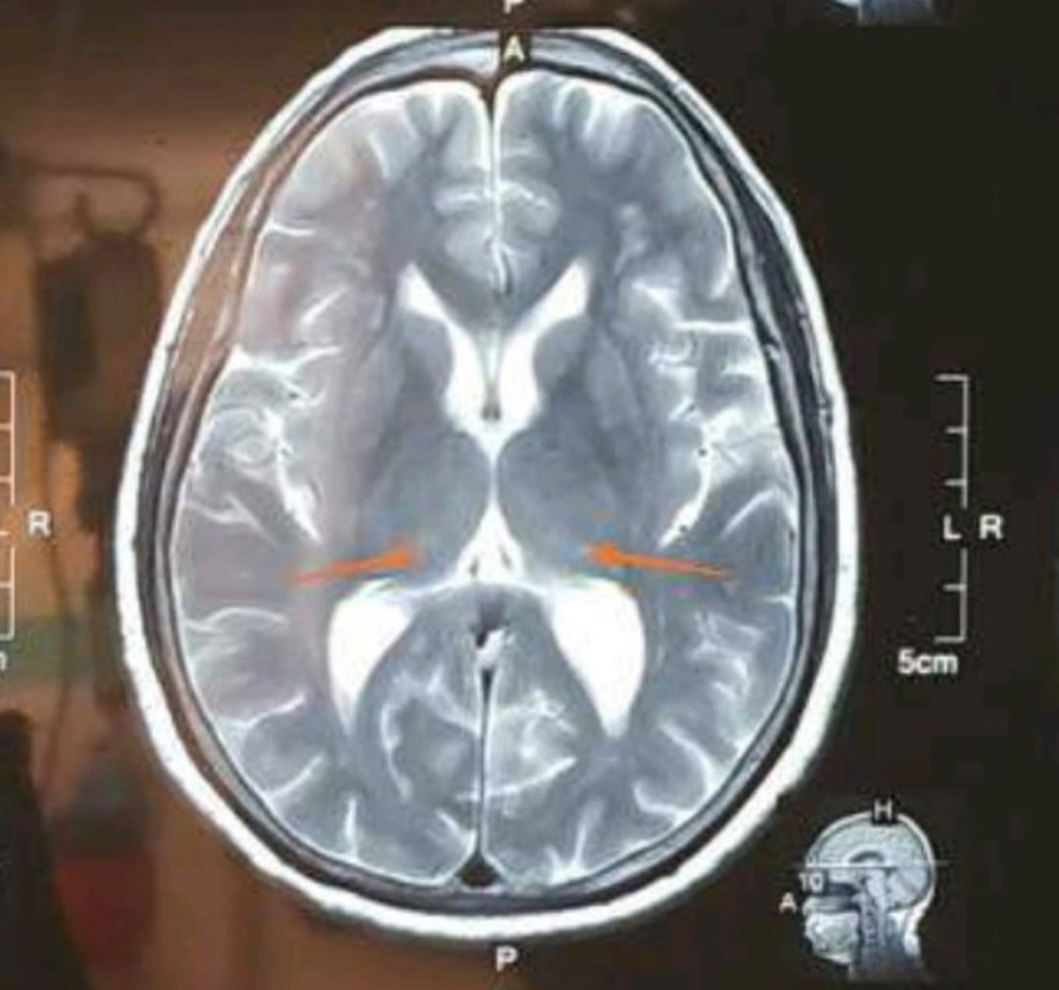
Subtle T2 high signal intensity in the pulvinar part of bilateral thalamus giving hockey‐stick appearance

MRI of the Brain revealed high signal intensity in bilateral frontal and occipital cortex as shown in Figure 2. Involvement of pulvinar nucleus of bilateral thalamus with subtle T2 high signal giving hockey stick appearance is also noted in our patient as shown in Figure 3.

Autoimmune encephalitis panel (NMDA, AMPA‐GluR1, AMPA‐GluR2, GABA‐B receptor antibody, LGl‐1 antibody, and CASPR2 antibody) was found to be negative.

The clinical features, progression of the disease course, EEG, and findings of brain imaging led us to the diagnosis of Creutzfeldt–Jakob disease. Our management protocol consisted of controlling seizures with a 3‐week course of sodium valproate and clobazam. We also carried out low volume plasma exchange for 5 days, during which the patient was infected with SARS‐CoV‐2 and shifted to the COVID building. The patient suffered from COVID pneumonia, and the condition worsened. However, the patient party left against medical advice due to financial constraints. The rest of the patient's condition was uneventful. The patient succumbed to death after 7 days that we confirmed via phone call.

## 3 DISCUSSION

sCJD is caused by spontaneous transformation of prion protein or through somatic mutation.^4^

Inherited CJD is associated with the mutation in human prion protein gene (PRNP).^4^ Iatrogenic CJD is extremely rare, reported to be caused by administration of cadaveric human pituitary hormone,^5^ dural graft transplants,^6^ and use of dural mater in radiological embolization procedures.^7^

The mean age for the onset of disease is around 62 years and with no one gender predominantly affected.^3^

CJD is clinically heterogeneous, with a common feature being rapid neuropsychiatric decline, with death occurring within one year of symptom onset. ^8^ The classic triad includes progressive dementia, myoclonus, and ataxia.^4^ Additional signs include behavioral abnormalities and deficits involving higher cortical function including aphasia, apraxia, and frontal lobe syndrome. Signs of extrapyramidal tract involvement such as hyperreflexia, extensor plantar responses (Babinski sign), and spasticity can be seen in about 40% of cases.^9^

Pathological studies of brain material to detect protease‐resistant PrPsc (PrPres) remain the gold standard for the diagnosis of prion disease. However, a probable diagnosis of sporadic CJD (sCJD) can be made with non‐invasive testing and is generally sufficient. According to the Centres for Disease Control and Prevention's criteria for the diagnosis of CJD, our patient fulfills the criteria for “probable” sporadic CJD.^10^ Our patient exhibited cognitive decline, ataxia, myoclonus, and akinetic mutism.

Hyperintense signal on DWI, FLAIR, and T2‐weighted images involving the cerebral cortex, corpus striatum, caudate head, and putamen is the most common pattern on MRI in patients with sporadic CJD.^11^, ^12^ Similarly, our patient on DWI has high signal intensity in bilateral frontal and occipital cortex. Involvement of pulvinar nucleus of bilateral thalamus with subtle T2 high signal giving hockey stick appearance is also noted in our patient.

A characteristic EEG pattern of CJD is periodic synchronous bi‐ or triphasic periodic sharp wave complexes (PSWC),^13^ which was present in our patient.

Additional investigations include real‐time quaking‐induced conversion, which has a sensitivity of 87–91 percent and specificity of 98–100 percent.^14^, ^15^ Detection of 14–3‐3 protein in CSF should be considered an adjunctive rather than diagnostic test for prion disease.^16^ CSF tau protein levels (>1150 picogram/mL) have superior accuracy and specificity as compared to 14–3‐3 protein as a diagnostic test.^17^ To the contrary, these tests could not be performed in our case due to financial constraints of the patient party.

The simultaneous clinical presentations of COVID‐19 and CJD in this patient may be due to absence of proper isolation facilities and frequent visitors visiting the patient during the covid surge in Nepal. The development of COVID pneumonia and worsening of further conditions have led us to hypothesize that the cascade of systemic inflammatory mediators that follow COVID‐19 may have hastened the prion disease pathogenesis and neurodegeneration. It may be due to the loss of region‐specific homeostatic identities of astrocytes and favoring neuroinflammatory transcriptional signatures. The molecular mechanism is not fully understood.

Other CSF studies such as S100 protein,^18^ Neuron‐specific enolase,^19^ and thymosin beta ^20^ have been reported in small series but not performed routinely due to low sensitivity and specificity.

Detecting abnormal prions outside CNS such as skin, nasal mucosa, skeletal muscle, peripheral nerves, and spleen has not been successful yet so as to recommend it as a diagnostic utility for clinical practice.^21^ Prion protein diagnostic assays performed using blood samples are in development.^22^

CJD is often mistaken for psychiatric disorders in the early course of disease as behavior and personality changes may be significant and obscure the accompanying cognitive features. However, with disease progression, neurologic signs become prominent, and an evaluation for dementia syndrome is generally undertaken. It must be differentiated from other dementias such as Alzheimer’s disease, dementia with Lewy bodies, and corticobasal degeneration, which are also associated with myoclonus and have a more rapid course. Other differential diagnoses include paraneoplastic syndrome and autoimmune encephalitis^23^ making neuroimaging and CSF analysis essential.

Before proceeding with the diagnosis, we ruled out many other vascular/ischemic, infectious, toxic/metabolic, auto‐immune, metastatic/neoplasm related, iatrogenic, systemic/ seizures/sarcoid, and demyelinating causes of rapidly progressive dementia,^24^on the basis of clinical presentation and best utilizing some investigations available in our setting. One of the differential diagnosis, autoimmune encephalitis, most commonly presents with early psychiatric manifestations and cognitive decline.^25^ Hashimoto's encephalopathy may have a relapsing–remitting course with stroke‐like symptoms; meanwhile, the course of sCJD is fulminant, leading to death within 1‐year time in 85% of patients.^26^ Symptoms typically present in vascular pathologies like headache and localizing motor; visual or sensory signs were absent in our case. She had no risk factors for vascular diseases as well as a normal coagulation profile. Cognition was not suddenly impaired as might be seen in strategic infarcts.^27^ The presence of akinetic mutism together with the classical EEG suggests that she presented to us in the late stage of the disease process.

There is no effective treatment for CJD, which is uniformly fatal, with a median disease duration of six months.^18^, ^28^ Supportive and symptomatic treatment is the only choice. Myoclonus may respond to benzodiazepines as well as to certain antiseizure medications such as levetiracetam and valproate.

Treatment with cholinesterase inhibitors or NMDA receptors antagonists are not useful and not used ^23^Potential therapies include flupirtine maleate, a centrally acting, non‐opioid analgesic that leads to upregulation of anti‐apoptotic protein Bcl‐2 displaying cytoprotective activity in vitro in neurons inoculated with a prion protein fragment.^29^ Pentosan polysulfide (PPS) is a heparin mimetic thought to interfere with the conversion of PrPc to PrPsc and should be given intraventricularly as it does not cross the blood–brain barrier.^30^

## 4 CONCLUSION

Rapidly progressing dementia is an interesting clinical scenario with a multitude of diagnostic possibilities. sCJD can be clinically heterogeneous, especially if initially presented with behavior and personality changes that obscures neurological findings. A thorough clinical assessment, diagnostic workup, and vigilance of primary health workers and psychiatrists are required to reach the diagnosis. Early diagnosis will allow patients and their families to prepare for the expected disease course, consider goals of care, and possibly have palliative care consultation if desired.

## AUTHOR CONTRIBUTIONS

Akash Raut, Anjila Thapa, Ashish Shrestha, Kamal saud, and Shailendra Katwal were involved in the writing of the manuscript. Dr.Reema Rajbhandari reviewed the manuscript. All the authors were involved in the final review of the manuscript.

## CONFLICT OF INTEREST

None.

## CONSENT

Written informed consent was obtained from the patient to publish this report in accordance with the journal's patient consent policy

## Data Availability

Our case report does not contain any data. However most of the reference articles are sited from pubmed.
